# NCGS like IBS ‘type’ symptoms is a diagnosis of exclusion

**DOI:** 10.1186/s12937-021-00737-x

**Published:** 2021-09-08

**Authors:** H. Ahmed, R. Hallam, G. Webster, A. Rej, I. D. Croall, S. H. Coleman, T. Key, R. Buckle, C. C. Shaw, J. Goodwin, I. Aziz, D. S. Sanders

**Affiliations:** 1 Academic Unit of Gastroenterology, Royal Hallamshire Hospital, Sheffield Teaching Hospital NHS Foundation Trust, Sheffield, S10 2JF UK; 2 Academic Unit of Radiology, University of Sheffield, Sheffield, UK

**Keywords:** NCGS, IBS, Gluten

Dear Editor,

We read with great interest the recent article by Moleski et al. [1], discussing symptoms of gluten ingestion in patients with Non-Celiac Gluten Sensitivity (NCGS). Notably, the authors highlight that without an objective marker for NCGS, participants within the study may have had alternative gastrointestinal diagnoses. Very little has been published about investigating patients with NCGS (that self-report symptoms occurring following the ingestion of gluten).

It is important to highlight to readers that patients who present with either self-reported NCGS or irritable bowel syndrome (IBS) ‘type’ symptoms should be investigated for other causes for their gastrointestinal symptoms. In view of this, we reviewed the case notes of patients with self-reported NCGS (*n* = 205) and with a new presentation of IBS ‘type’ symptoms (*n* = 74). Patients were divided into two groups, group one consisted of individuals presenting with self-reported NCGS. In contrast, group two consisted of individuals with a new presentation of IBS ‘type’ symptoms. Individuals were characterised according to demographics, in addition to investigative outcomes.

We found that patients presenting with NCGS and IBS ‘type’ symptoms were predominantly female, at 84% (*n* = 172) and 62% (*n* = 46) respectively. There was no significant difference in the presenting age between patients (NCGS mean age 40.0 ± 15.7 years vs IBS mean age 38.4 ± 14.1 years; *p* = 0.56). Following investigation, 11.7% (*n* = 24) of NCGS and 17.6% (*n* = 13) of patients with IBS ‘type’ symptoms were found to have other gastrointestinal diagnosis (Tables 1 and 2). The most common diagnosis for the NCGS group was celiac disease (CD) (8.8%, *n* = 18) and by comparison for patients with IBS ‘type’ symptoms it was bile acid diarrhoea (BAD) (13.5%, *n* = 10).

**Table 1:** Breakdown of the diagnosis of patients presenting with self-reported gluten sensitivity

**Final primary diagnosis**	**Number of patients (%)**
Non-celiac gluten sensitivity (NCGS)	181 (88.3)
Celiac disease (CD)	18 (8.8)
Fructose intolerance	1 (0.5)
Lactose intolerance	2 (1.0)
Small intestinal bacterial overgrowth (SIBO)	3 (1.5)
**Total**	205 (100.0)

**Table 2:** Breakdown of the diagnosis of patients presenting with IBS ‘type’ symptoms

**Final primary diagnosis**	**Number of patients (%)**
Irritable Bowel Syndrome (IBS)	61 (82.4)
Bile acid diarrhoea (BAD)	10 (13.5)
Microscopic colitis	2 (2.7)
Pancreatic exocrine insufficiency (PEI)	1 (1.4)
**Total**	74 (100.0)

We conclude that the demographics of patients with NCGS and IBS are similar, with a young female presentation. We found a significant minority of these patients have other GI diagnoses. The data supports the recommendation that patients presenting with self-reported NCGS should only be confirmed as having NCGS after excluding other gastrointestinal disorders. We have also demonstrated from our data that a similar approach is required in patients with IBS ‘type’ symptoms. One strategy that may help would be the utilisation of the Salerno experts’ criteria in reaching a firm and positive diagnosis of NCGS which would in turn improve the validity of the results published [2]. Whilst this approach may be regarded as cumbersome, it would facilitate comparison of different studies if adopted universally [2]. We fear that until a validated a biomarker of NCGS is discovered, further research in NCGS pathogenesis and triggers may be futile.

## Data Availability

N/A
